# Multiscale Prediction of Heat and Mass Transport Properties in Cement-Based Materials Based on Hydration Microstructure Evolution

**DOI:** 10.3390/ma19143087

**Published:** 2026-07-17

**Authors:** Fali Huang, Zhenhao Wang, Chenyun Yu, Bin Peng, Fengjuan Wang, Zhiqiang Yang, Yuncheng Wang

**Affiliations:** 1 State Key Laboratory of High-Speed Railway Track System, Beijing 100081, China; 18811789830@126.com (F.H.);; 2 Railway Engineering Research Institute, China Academy of Railway Science Corporation Limited, Beijing 100081, China; 3 State Key Laboratory of Engineering Materials for Major Infrastructure, Southeast University, Nanjing 211189, China; wzh18738452730@163.com (Z.W.);; 4 School of Materials Science and Engineering, Southeast University, Nanjing 211189, China; 5 China Railway Engineering Consulting Group Co., Ltd., Beijing 100071, China

**Keywords:** cement-based materials, DTW-CEMHYD3D, Mori–Tanaka homogenization, heat and mass transport coefficients, hydration microstructure

## Abstract

This study proposes a multiscale prediction method that couples the DTW-CEMHYD3D hydration kinetics model with Mori–Tanaka homogenization theory to establish a quantitative relationship between hydration microstructure evolution and macroscopic heat and mass transport properties of cement-based materials. First, dynamic time warping (DTW) is introduced to correct the mapping relationship between the hydration cycle number in CEMHYD3D and the real hydration time. Then, cement-based materials are regarded as multiphase composites, and a unified calculation method for the effective diffusion coefficient and thermal conductivity is established. The results show that the DTW-CEMHYD3D model can markedly improve the prediction accuracy of early-age hydration heat. The average relative error between the predicted effective diffusion coefficient and the N-phase sphere model is 0.67%, while the coefficients of determination for thermal conductivity prediction and relative diffusion coefficient prediction during hydration reach 0.9812 and 0.987, respectively. Parametric analysis indicates that a higher water-to-binder ratio significantly increases the relative diffusion coefficient, and this effect exhibits nonlinear enhancement with hydration age. The influence of fly ash content is age-dependent. An increase in the degree of saturation reduces the chloride diffusion coefficient but only slightly increases the thermal conductivity. The proposed method provides a reference for durability analysis and parameter determination in multiphysics coupling models of cement-based materials.

## 1. Introduction

Cement-based materials are among the most widely used porous composites in civil engineering, and their service performance is closely related to the formation of hydration products, the evolution of pore structure, and multiphase transport processes. During cement hydration, unhydrated particles gradually dissolve and generate hydration products such as calcium silicate hydrate (C-S-H) gel and calcium hydroxide, causing the microstructure to evolve from an initial particle-packing system into a complex multiphase system composed of a solid skeleton, pore solution, and multiscale pores [1,2,3]. This process not only governs strength development but also directly affects the transport capacity of ions, moisture, and heat within the pore system [4,5]. In service environments such as chloride attack [6], sulfate attack [7], and freeze–thaw cycles [8], the durability degradation of cement-based materials is strongly influenced by the hydration microstructure. Therefore, establishing a multiscale prediction method that reflects the relationship between hydration microstructure evolution and macroscopic heat and mass transport properties is of great significance for durability assessment and service performance prediction of cement-based materials.

Early studies on the microstructure of cement-based materials mainly relied on characterization techniques such as mercury intrusion porosimetry (MIP) [9] and scanning electron microscopy (SEM) [10,11]. These methods have been widely used to characterize the degree of hydration, porosity, and pore size distribution of cement paste [12,13]. They also provide an indirect basis for relating microstructural features to mechanical and mass transport properties. However, such methods have several limitations. On the one hand, experimental characterization is time-consuming and labor-intensive, and the inherent randomness of the concrete microstructure makes it difficult to ensure specimen homogeneity, leading to relatively poor test repeatability. On the other hand, experimental results generally reflect only the static structure at a specific time, making it difficult to dynamically track the evolution of structure and properties during hydration or to capture the transitional behavior at key hydration ages, such as 12 h, 24 h, and 72 h. This limitation is particularly important because the freeze–thaw damage of cement-based materials exposed to cold regions at early ages is highly sensitive to the structural stability during this stage [14].

On this basis, numerical simulation techniques have been gradually developed to describe the microstructural evolution and microscopic properties of cement-based materials. The earliest computer-based study on cement hydration can be traced back to the single-component mineral phase simulation model proposed by Pommersheim et al. [15]. Jennings et al. [16] subsequently introduced computer-based digital image technology, pioneering the visual simulation of hydration processes and microstructural evolution. Over the following three decades, several mature numerical models were developed, such as the DuCOM model [17], the CEMHYD3D hydration model [18,19,20], the HymoStruc3D model [21], and the μic hydration model [22]. According to their working principles, these models can be generally classified into continuum-based models and digital-image-based models. Among them, the CEMHYD3D model has become one of the most widely used hydration kinetics models because of its higher spatial resolution and its ability to visually represent the three-dimensional evolution of cement hydration [23,24,25]. However, the classical CEMHYD3D model adopts an empirical relationship between simulation cycle number and hydration time, which may still lead to deviations in predicting the heat release process during early hydration, especially in the dissolution and induction periods. Moreover, in blended cementitious systems containing mineral admixtures such as fly ash, the early-age reaction rate, heat release characteristics, and time-dependent response become more complex, making the classical time-mapping approach less adaptable to hydration processes under different mix proportions [25]. Therefore, the time-mapping relationship in CEMHYD3D should be modified to improve the prediction accuracy of early-age hydration heat and subsequent microstructural evolution.

In addition to the hydration microstructure itself, transport parameters such as the effective diffusion coefficient [26], thermal conductivity [27], and permeability [28] of cement-based materials are also key inputs for durability models. In existing models for chloride diffusion, hygrothermal coupling, freeze–thaw damage, and multiphysics coupling, transport parameters are commonly obtained from empirical formulas, experimental fitting, or fixed values. These approaches have difficulty reflecting the combined effects of the water-to-binder ratio (w/b), mineral admixtures, hydration age, and pore saturation degree on transport properties [7,29,30,31]. On the other hand, cement-based materials exhibit typical multiphase composite characteristics, and their macroscopic transport properties are essentially governed by microstructural constituents such as hydration products, unhydrated particles, pore phases, and pore solution. Relying solely on macroscopic experimental parameters makes it difficult to reveal the intrinsic relationship between microstructure and heat and mass transport properties [32,33]. To quantitatively describe the cross-scale relationship between microstructure and macroscopic heat and mass transport coefficients, homogenization theory provides an effective theoretical framework [34,35,36,37]. In particular, Mori–Tanaka homogenization was originally developed to estimate the effective elastic modulus of composites, and it also provides a theoretical basis for multiscale prediction from hydration microstructure to macroscopic heat and mass transport properties [37]. However, the effective coupling of hydration kinetics modeling with homogenization theory and the resulting influence of microstructure on heat and mass transport coefficients require further investigation.

Although previous studies have combined hydration models with homogenization methods to predict transport properties, two limitations remain. First, the time-mapping relationship in classical CEMHYD3D is usually empirical, which may reduce the accuracy of early-age hydration prediction, especially for blended cementitious systems containing fly ash. Second, the prediction of transport properties is still highly dependent on the accuracy of hydration simulation, whereas the influence of hydration-model uncertainty on subsequent multiscale transport prediction has received limited attention.

To address these gaps, this study establishes a multiscale prediction method for heat and mass transport coefficients in cement-based materials by coupling DTW-corrected CEMHYD3D hydration kinetics with Mori–Tanaka homogenization theory. First, dynamic time warping (DTW) is introduced to modify the hydration time-mapping relationship in the CEMHYD3D model, thereby forming the DTW-CEMHYD3D hydration kinetics model. Second, calculation methods for the effective diffusion coefficient, thermal conductivity, and permeability are derived based on Mori–Tanaka homogenization, and the results obtained from the DTW-CEMHYD3D hydration kinetics model are coupled into the calculation framework. Finally, the effectiveness of the proposed model is verified using several cases, and the influence mechanisms of the water-to-binder ratio, fly ash content, and pore saturation degree on heat and mass transport properties are analyzed. This study provides a microstructure-driven prediction approach for durability analysis and parameter determination in multiphysics coupling models of cement-based materials.

## 2. Hydration Kinetics Model and Microstructure Characterization

### 2.1. DTW-CEMHYD3D Hydration Kinetics Model

To obtain the microstructural evolution of cement-based materials during hydration, the CEMHYD3D hydration kinetics model, which is based on the cellular automaton principle, was adopted to simulate the hydration process of cementitious systems. The basic computational procedure of the CEMHYD3D (Version 3.0) model includes raw material parameter input, particle generation and spatial discretization, phase assignment, hydration reaction simulation, and microstructure post-processing. The CEMHYD3D model has been widely used, and more detailed computational procedures can be found in the relevant literature.

In the classical CEMHYD3D model, the hydration time factor plays an important role in determining the shape of the hydration heat curve. As discussed in the Introduction, the prediction accuracy and applicability of the classical CEMHYD3D model still need to be improved for early-age hydration and blended cementitious systems. To improve the simulation accuracy of CEMHYD3D for early-age hydration, DTW was introduced in this study, and hydration heat test results were used to correct the time-mapping relationship of the model. Accordingly, a DTW-CEMHYD3D hydration kinetics model was established.

DTW is used to measure the similarity between two time series. Its core objective is to identify the optimal matching path between two sequences. Even when stretching or shifting exists along the time axis, DTW can align the sequences through nonlinear time transformation and thereby quantify their similarity.

The two time series are defined as follows:

(1) $$\begin{array}{l} X=\left(x_{1},x_{2},\ldots ,x_{n}\right)\\ Y=\left(y_{1},y_{2},\ldots ,y_{m}\right) \end{array}$$

where *n* and *m* denote the lengths of the two sequences, respectively.

An *n* × *m* distance matrix *D* is first constructed, in which *D*(*i*, *j*) represents the distance between *x*_i_ and *y*_j_. This distance is usually calculated using the Euclidean distance as follows:

(2) $$D\left(i,j\right)={\left(x_{i}-y_{i}\right)}^{2}$$

Subsequently, the cumulative distance matrix *C* is constructed. Each element *C*(*i*, *j*) in this matrix represents the minimum cumulative distance from the starting point (1, 1) of the sequences to the point (*i*, *j*). The matrix *C* is calculated using dynamic programming according to the following recursive formula:

(3) C(i,j)=D(i,j)+min(C(i−1,j),C(i,j−1),C(i−1,j−1))

where *C*(1,1) = *D*(1, 1) is taken as the initial condition.

After the calculation is completed, backtracking starts from (C(*n*, *m*)). At each step, the direction with the minimum cumulative distance among the three adjacent cells is selected until the path returns to ((1, 1)). In this way, the optimal matching path between the two time series can be obtained. The DTW distance is then defined as the cumulative distance along this optimal path, namely (C(*n*, *m*)), which provides a quantitative measure of the similarity between the two sequences. To improve computational efficiency and avoid unreasonable matching, certain constraints are usually introduced. The most commonly used constraint is the Sakoe–Chiba band, which limits the extent to which the matching path can deviate from the diagonal. This constraint can reduce computational complexity and prevent excessive time warping.

In the CEMHYD3D calculation, the relationship between the hydration cycle number and hydration heat was obtained and used as the first time series in DTW:

(4) $$H_{\mathrm{Sim}}=\left(h_{1}^{s},h_{2}^{s}\ldots ,h_{n}^{s}\right)$$

where *H*_Sim_ denotes the cumulative hydration heat released during the entire simulation process, and *h*_i_*^s^* represents the cumulative heat release at the *i*-th simulation cycle.

Meanwhile, the relationship between hydration time and hydration heat obtained from the experimental test was used as the second time series in DTW:

(5) $$H_{\mathrm{Exp}}=\left(h_{1}^{e},h_{2}^{e}\ldots ,h_{m}^{e}\right)$$

where *H*_Exp_ denotes the cumulative hydration heat released during the entire experimental process, and *h*_i_*^e^* represents the cumulative heat release at the *i*-th experimental time point.

By matching *H*_Sim_ and *H*_Exp_ using the DTW algorithm, a nonlinear mapping relationship between the simulation cycle number in CEMHYD3D and the real hydration time can be established. This enables dynamic correction of the hydration time factor in the CEMHYD3D model.

The DTW mapping is established by minimizing the cumulative distance between the simulated hydration heat curve generated by CEMHYD3D and the corresponding experimental hydration heat curve, thereby providing a nonlinear correspondence between simulation cycles and real hydration time. Since the hydration kinetics are influenced by the binder composition and curing conditions, the DTW mapping is not regarded as a universal time-mapping function. For cementitious systems with significantly different binder compositions or curing conditions, recalibration of the DTW mapping is generally recommended to maintain prediction accuracy. In addition, the present DTW correction is primarily intended for interpolation within the calibration range covered by the experimental data, whereas its applicability beyond this range requires further investigation.

### 2.2. Dynamic Evolution of Hydration Microstructure and Hydration Product Content

#### 2.2.1. Dynamic Evolution of Hydration Microstructure

**Figure 1:**
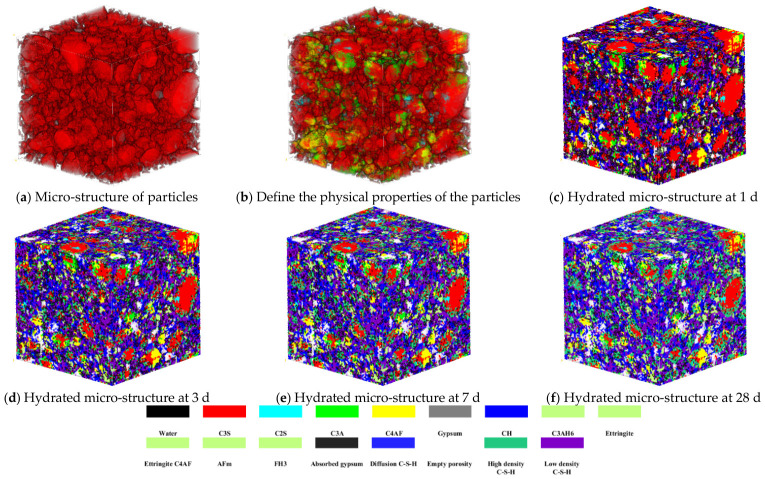
Evolution of hydration microstructure of 0.40 w/b cement paste.

**Figure 2:**
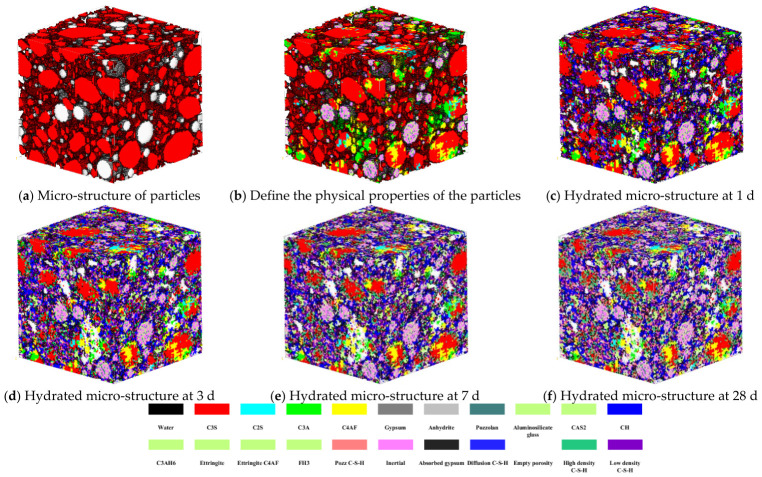
Evolution of hydration microstructure of 20% fly ash paste.

Based on the DTW-CEMHYD3D hydration kinetics model, three-dimensional hydration microstructures of cementitious systems with different water-to-binder ratios, fly ash contents, and hydration ages can be obtained. Figure 1 and Figure 2 show the hydration microstructures of cement paste with a typical water-to-binder ratio and paste with 20% fly ash content at different hydration ages, respectively.

The evolution of the hydration microstructure shows that, as hydration proceeds, the phases around the particles gradually dissolve, and free water in the microstructure is continuously consumed. Meanwhile, a large amount of hydration products is generated and progressively fills the interparticle pores. Hydration products preferentially nucleate and grow on particle surfaces, which is more evident on the surfaces of small cement particles. This indicates that particles with a larger specific surface area are more prone to hydration and nucleation reactions. With increasing hydration age, C-S-H gel gradually interconnects to form a network-like structure and penetrates the spaces between unhydrated particles, providing a basis for the continuity and densification of the microstructure.

#### 2.2.2. Dynamic Evolution of Hydration Product Content

To further quantify the phase changes during hydration, the phase volume fractions of cementitious systems at different hydration ages were extracted based on the DTW-CEMHYD3D model. The accuracy of hydration product quantification using the same type of CEMHYD3D hydration kinetics model has been verified in previous studies [38,39,40,41]. Since no new hydration reaction system or hydration product was introduced in this study, this validation was not repeated.

**Figure 3:**
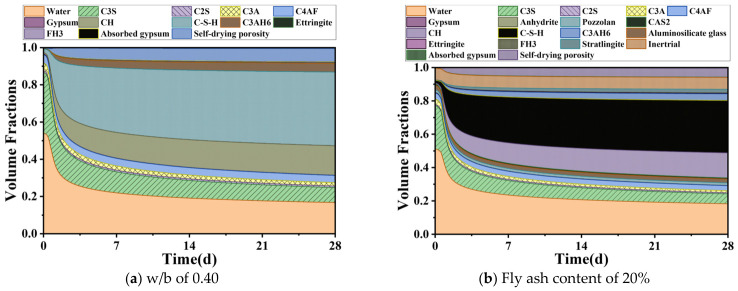
Quantitative analysis of the volume fraction of hydrated microstructures.

Figure 3 shows the evolution of phase volume fractions in two representative systems. For the cement paste with a w/b of 0.40, C3S was significantly consumed within the first 7 d, with its volume fraction decreasing from approximately 35% initially to less than 10%. The volume fraction of C-S-H increased rapidly with hydration age, and its growth rate gradually slowed after 7 d. The volume fraction of CH continued to increase and reached approximately 15% at 28 d. Meanwhile, the volume fraction of free water continuously decreased as hydration proceeded, while approximately 15% remained at 28 d. Self-desiccation pores gradually formed with increasing hydration age and reached approximately 20% at 28 d.

For the FA20% composite system, the phase composition changed after the incorporation of fly ash. The results show that the system containing 20% fly ash had a higher C-S-H content and a lower CH content at 28 d, indicating that fly ash participated in the later-age hydration reaction and modified the composition of hydration products. Meanwhile, the self-desiccation porosity of the FA20% system was approximately 5% at 28 d, which was lower than that of systems with lower fly ash contents. This indicates that pore filling in this system was enhanced during the middle and later stages of hydration. Overall, both the cement pastes with a w/b of 0.40 and the FA20% composite system showed similar features with increasing hydration age, namely an increase in hydration products, a decrease in free water, and gradual filling of pore space.

Based on the DTW-CEMHYD3D model, the phase volume fractions of unhydrated particles, hydration products, free water, and pores at different hydration ages can be obtained. In Section 3, these phase volume fractions are used as inputs for the homogenization model, in which cement-based materials are regarded as multiphase composites composed of a matrix phase and multiple inclusion phases, and their effective heat and mass transport coefficients are calculated.

## 3. Multiscale Prediction Model for Heat and Mass Transport Coefficients Based on Homogenization Theory

### 3.1. Equivalent Assumptions for Multiphase Composites

As typical multiphase composites, the macroscopic transport properties of cement-based materials, including the diffusion coefficient, thermal conductivity, and permeability, are essentially governed by their microstructure. During hydration, the original cement particles are gradually surrounded by hydration products, forming a complex multiphase microstructure. The evolution of this multiphase structure directly affects ion transport paths, heat conduction paths, and fluid permeation paths. Therefore, it is necessary to establish a quantitative relationship between microstructural parameters and macroscopic transport coefficients. In this section, based on micromechanics theory, the phase volume fractions of the composite are considered, and the Mori–Tanaka method [37] is adopted to predict the effective heat and mass transport coefficients of cement-based materials under different conditions.

**Figure 4:**
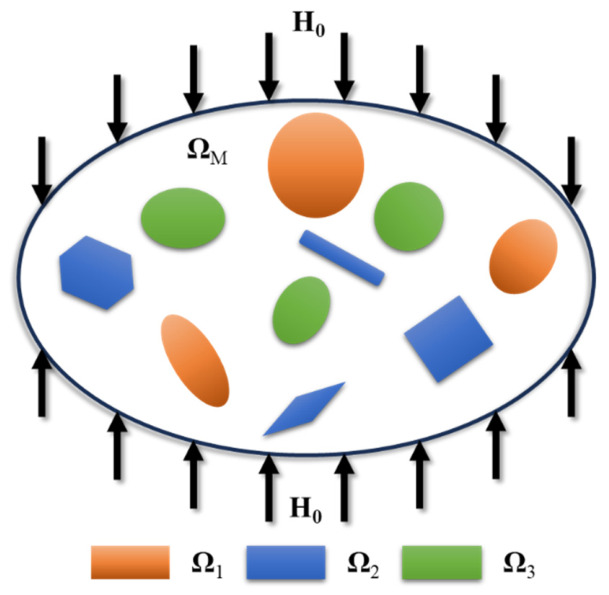
Schematic representation of a representative volume element for a multiphase composite, including the matrix phase Ω_M_ and multiple inclusion domains Ω_1_, Ω_2_, and Ω_3_; the scheme can be extended to systems containing more phases.

From the perspective of aggressive medium transport and energy transfer, the prediction of various effective transport parameters, such as thermal/electrical conductivity, permeability, and diffusivity, is mathematically similar [42]. This problem is usually analyzed using a representative volume element Ω (RVE) of a statistically homogeneous material. The material is assumed to consist of an infinite homogeneous matrix Ω_M_ and a multi-inclusion system Ω_inc_, as shown in Figure 4.

During heat and mass transport, the properties of each phase are assumed to be uniformly distributed within that phase, and the inclusions are assumed to be randomly distributed in the matrix. The overall material is statistically homogeneous, the interfaces are perfectly bonded without interfacial resistance, and the transport process is treated as quasi-static, with inertial effects neglected. These assumptions allow the complex hydration microstructure to be treated as an equivalent multiphase medium and make the Mori–Tanaka calculation analytically tractable. However, they also imply that local transport heterogeneity is simplified. In real cementitious materials, interfacial transition zones may have transport properties different from those of the bulk matrix, pore connectivity may be direction-dependent, and cracks or microcracks may provide preferential transport pathways. Therefore, the predicted effective coefficients should be interpreted as homogenized transport parameters for statistically averaged microstructures rather than as local transport properties at specific interfaces or cracked regions.

In Ω_M_, the volume fraction and diffusion coefficient of the matrix are denoted as *V*_m_ and *D*_m_, respectively. In Ω_inc_, the volume fraction and diffusion coefficient of each inclusion phase are denoted as *V_r_* and *D_r_*, respectively, where *r* = 1, 2, …, *M*, and *M* is the number of inclusion types. Inclusions with the same physical properties are regarded as one type, although they may have different sizes and morphologies. Accordingly, the following relationship among the phases can be obtained:

(6) $$V_{m}+V_{1}+V_{2}+\ldots +V_{M}=1$$

This constraint ensures the conservation of phase volume fractions and serves as the basis for the subsequent homogenization derivation.

### 3.2. Effective Heat and Mass Transport Coefficients for a Single Inclusion

The theoretical formulations in this section are mainly based on established micromechanical theories, including Eshelby’s equivalent inclusion theory, concentration tensor theory, and Mori–Tanaka homogenization. These equations are adopted or adapted from the literature to construct a unified transport-parameter calculation framework, rather than newly proposed as fundamental micromechanical theories in this work.

Taking ion diffusion in a single inclusion as an example, **H** and **J** are first defined as the intensity field and diffusion flux, respectively. According to the flux conservation law in the transport process, the relationship between the diffusion flux and the concentration gradient in each phase can be expressed as:

(7) $$J_{i}=D_{ij}H_{j}=-D_{ij}\frac{\partial \varphi }{\partial x_{j}}$$

(8) ΔJ=0

where **J***_i_* is the diffusion flux of phase *i*, with (*i* = *m*, 1, 2, 3, …, *N*); **H***_j_* is the concentration gradient; and the negative sign indicates that mass diffusion occurs in the direction opposite to the concentration gradient. **D***_ij_* is the second-order tensor of the diffusion coefficient. For heat transfer, **D***_ij_* can correspond to the thermal conductivity tensor, while for permeation, it can correspond to the permeability tensor. Therefore, this mathematical form can be used to uniformly describe ion diffusion, heat conduction, and fluid permeation. *x_j_* denotes the coordinate direction, where (*j* = 1, 2, 3) corresponds to the *x*, *y*, and *z* directions, respectively. *φ* is the volume concentration in the matrix. For heat transfer, *φ* represents temperature, while for permeation, it represents pressure potential.

For the averaging treatment of boundary conditions, a uniform intensity boundary condition **H**_0_ is applied to the exterior of the material element, so that it can approximate the in situ state of the internal material element as closely as possible. The corresponding boundary condition can be expressed as

(9) $$\oint_{S}\varphi \left(x\right)n_{i}dS=H_{0}\cdot S$$

where *S* is the area of the outer surface, **H**_0_ is the overall intensity, and *n*_i_ is the *i*-th component of the unit outward normal vector on the outer surface.

In the present system, multiphase inclusions are randomly distributed in an infinite matrix; therefore, the representative volume element can be regarded as a statistically homogeneous medium. Since the inclusions do not exhibit long-range ordering, the macroscopic properties can be considered as the statistical average of the contributions from microscopic elements. The volume-averaged intensity and volume-averaged diffusion flux can be expressed as:

(10) $$\langle H\rangle =\frac{1}{\Omega }\int_{\Omega }H_{i}\left(x\right)d\Omega =V_{m}\langle H_{m}\rangle +\sum_{r=1}^{N}V_{r}\langle H_{r}\rangle$$

(11) $$\langle J\rangle =\frac{1}{\Omega }\int_{\Omega }J_{i}\left(x\right)d\Omega =V_{m}\langle J_{m}\rangle +\sum_{r=1}^{N}V_{r}\langle J_{r}\rangle$$

where $\langle H\rangle$ is the local intensity in the matrix, $\langle H_{r}\rangle$ is the local intensity in inclusion phase *r*, $\langle J_{m}\rangle$ is the local diffusion flux in the matrix, and $\langle J_{r}\rangle$ is the local diffusion flux in inclusion phase *r*. *V*_m_ and *V*_r_ are the volume fractions of the matrix and inclusion phase *r*, respectively.

The above volume-averaging operation converts local field variables into macroscopic equivalent quantities, serving as a key bridge from the microscale to the macroscale. According to the Mori–Tanaka mean-field theory [37], the constitutive relationships of single-phase and porous composite materials can be expressed as:

(12) $$\langle J\rangle =-D_{ij}^{eff}\langle H\rangle =-D_{ij}^{eff}H_{0}$$

(13) $$\langle J_{m}\rangle =D_{m}\langle H_{m}\rangle$$

(14) $$\langle J_{r}\rangle =D_{r}\langle H_{r}\rangle$$

where $D_{ij}^{eff}$ is the overall effective diffusion coefficient of the composite material, representing the macroscopic equivalent parameter obtained by homogenizing the microscale inclusions and matrix. Combining the above equations gives:

(15) $$D_{eff}H_{0}=D_{m}V_{m}\langle H_{m}\rangle +\sum_{r=1}^{N}D_{r}V_{r}\langle H_{r}\rangle$$

(16) $$D_{eff}=D_{m}+\sum_{r=1}^{N}\left(D_{r}-D_{m}\right)V_{r}\cdot \frac{\langle H_{r}\rangle }{H_{0}}$$

where ${\langle H_{r}\rangle /H}_{0}$ is the intensity amplification factor, which reflects the amplification or attenuation of the local intensity relative to the applied intensity. The spatial distribution of this ratio directly determines the effective transport coefficient and is influenced by factors such as the inclusion shape.

To describe the linear relationship between the local intensity field and the applied intensity field, the second-order concentration tensor **A**^(*r*)^ is introduced to achieve micro-to-macro multiscale coupling [43], as follows:

(17) $$\langle H_{r}\rangle =A^{\left(r\right)}\cdot H_{0}$$

Based on Eshelby’s equivalent inclusion theory [44], the following established relationship between the internal intensity and the applied intensity for a single ellipsoidal inclusion embedded in an infinite matrix is adopted:

(18) $${\langle H_{r}\rangle }^{\left(1\right)}=A^{\left(1\right)}\cdot H_{0}$$

where the superscript ^(1)^ denotes a single inclusion, and **A**^(1)^ represents the perturbation of the intensity field caused by the single inclusion. It is expressed as

(19) $$A^{\left(1\right)}={\left[I+S\left(\frac{D_{r}}{D_{m}}-I\right)\right]}^{-1}$$

where **S** is the depolarization factor [45], which describes the influence of the inclusion shape on the distribution of the surrounding intensity field. This factor is determined only by the geometry of the inclusion and is independent of the material properties. For a standard spherical inclusion, **S** = *δ*_ij_/3. For ellipsoidal or other non-spherical inclusions, it can be calculated as follows:

(20) $$S=\left(\begin{array}{ccc} Q & 0 & 0\\ 0 & Q & 0\\ 0 & 0 & 1-2Q \end{array}\right)$$

(21) $$Q=\left\{\begin{array}{c} \frac{1}{2}\left\{1+\frac{1}{\kappa^{2}-1}\left[1-\frac{\kappa }{2\sqrt{\kappa^{2}-1}}\ln\left(\frac{\kappa +\sqrt{\kappa^{2}-1}}{\kappa -\sqrt{\kappa^{2}-1}}\right)\right]\right\}\;,\;\kappa \;>\;1\\ \frac{1}{2}\left\{1+\frac{1}{\kappa^{2}-1}\left[1-\frac{\kappa }{\sqrt{1-\kappa^{2}}}\arctan\left(\frac{\sqrt{1-\kappa^{2}}}{\kappa }\right)\right]\right\}\;,\;\kappa \;>\;1 \end{array}\right.$$

where *κ* is the aspect ratio of the inclusion, defined as *κ = a/b*. When *κ* > 1, the inclusion is a prolate spheroid; when *κ* < 1, the inclusion is an oblate spheroid. In the present formulation, **S** denotes the depolarization tensor, whereas *Q* is the scalar shape factor used for isotropic averaging of ellipsoidal inclusions. For spherical inclusions, *Q* = 1/3; for fiber-like inclusions, *Q* = 1/2; and for plate-like inclusions, *Q* = 0. To avoid ambiguity, the same notation is used consistently in the subsequent validation cases.

The depolarization factor and the corresponding shape factor are geometric parameters that describe how the inclusion morphology influences the redistribution of the transport field within the surrounding matrix. The aspect ratio characterizes the inclusion geometry, whereas the concentration tensor determines the relationship between the local transport intensity inside the inclusion and the externally applied field. Together, these parameters govern the effective transport response predicted by the homogenization framework.

When the inclusions have a single morphology, **A**^(*r*)^ = **A**^(1)^. Accordingly, the expressions for the ionic diffusion coefficient, thermal conductivity, and permeability under the single-inclusion condition can be derived as follows:

(22) $$D_{eff}=D_{m}+\sum_{r=1}^{N}\left(D_{r}-D_{m}\right)V_{r}\cdot A^{\left(1\right)}=V_{m}D_{m}+V_{1}D_{1}A^{\left(1\right)}$$

(23) $$k_{eff}=k_{m}+\sum_{r=1}^{N}\left(k_{r}-k_{m}\right)V_{r}\cdot A^{\left(1\right)}=V_{m}k_{m}+V_{1}k_{1}A^{\left(1\right)}$$

(24) $$K_{eff}=K_{m}+\sum_{r=1}^{N}\left(K_{r}-K_{m}\right)V_{r}\cdot A_{r}=V_{m}K_{m}+V_{1}K_{1}A^{\left(1\right)}$$

where *k*_eff_ is the effective thermal conductivity of the composite material, *K*_eff_ is the effective permeability of the composite material, *k*_m_ is the thermal conductivity of the matrix, and *K*_m_ is the permeability of the matrix.

The above single-inclusion model provides the basic theoretical unit for calculating the effective heat and mass transport coefficients of subsequent multiphase inclusion systems.

### 3.3. Effective Heat and Mass Transport Coefficients for Multiphase Inclusions

Cement-based materials contain various types of inclusions, such as pores, interfacial transition zones, aggregates, hydration products, and unhydrated particles, which have different geometrical and physical properties. Therefore, the single-inclusion case needs to be extended to a multiphase inclusion system.

In the multiphase inclusion model, the interactions among inclusions are considered, and the equivalent uniform field intensity **H***_im_* is introduced to replace the overall intensity **H**_0_. The relationship between the intensity of each phase and the virtual intensity **H***_im_* can be expressed as [46]

(25) $$\langle H_{r}\rangle =A^{\left(r\right)}\cdot H_{im}\;,\;\langle H_{m}\rangle =H_{im}$$

where **A**^(*r*)^ is the concentration factor of inclusion phase *r*, which can be approximately obtained from the single-inclusion formula. According to the preceding relationship, the virtual intensity **H***_im_* can be expressed as

(26) $$H_{im}={\left[V_{m}\cdot I+\sum_{r=1}^{N}V_{r}\cdot A^{\left(r\right)}\right]}^{-1}H_{0}$$

Substituting Equation (25) into Equation (26) gives the expression of the concentration factor **A***^eff^* in the mean-field theory as follows:

(27) $$A^{eff}=A^{\left(r\right)}{\left[V_{m}\cdot I+\sum_{r=1}^{N}V_{r}\cdot A^{\left(r\right)}\right]}^{-1}$$

By replacing **A**^(*r*)^ in Equation (17) with **A***^eff^*, the effective diffusion coefficient based on the mean-field theory can be expressed as

(28) $$D_{eff}=D_{m}+\sum_{r=1}^{N}\left(D_{r}-D_{m}\right)V_{r}\cdot A^{\left(r\right)}{\left[V_{m}\cdot I+\sum_{r=1}^{N}V_{r}\cdot A^{\left(r\right)}\right]}^{-1}$$

For composites with randomly oriented inclusions, the effective diffusion coefficient can be regarded as isotropic. Accordingly, the average effective diffusion coefficient of the composite can be obtained from Equations (19), (27) and (28) as:

(29) $$\langle D_{eff}\rangle =D_{m}+\left[\sum_{r=1}^{N}\left(D_{r}-D_{m}\right)V_{r}\cdot \langle A^{\left(r\right)}\rangle \right]{\left[V_{m}+\sum_{r=1}^{N}V_{r}\cdot \langle A^{\left(r\right)}\rangle \right]}^{-1}$$

(30) $$\langle A^{\left(r\right)}\rangle =\frac{1}{3}\left[\frac{2D_{m}}{\left(1-Q_{r}\right)D_{m}+Q_{r}D_{r}}+\frac{D_{m}}{2Q_{r}D_{m}+\left(1-2Q_{r}\right)D_{r}}\right]$$

where *Q*_r_ is an intermediate variable used to calculate the depolarization factor of inclusion phase *r*, and it can be obtained from Equation (21).

Equations (29) and (30) provide the micromechanical model for multiple inclusions. This model can be used not only to derive the average effective diffusion coefficient of multiphase composites, but also to calculate the average effective thermal conductivity and average effective permeability:

(31) $$\langle k_{eff}\rangle =k_{m}+\left[\sum_{r=1}^{N}\left(k_{r}-k_{m}\right)V_{r}\cdot \langle A^{\left(r\right)}\rangle \right]{\left[V_{m}+\sum_{r=1}^{N}V_{r}\cdot \langle A^{\left(r\right)}\rangle \right]}^{-1}$$

(32) $$\langle K_{eff}\rangle =K_{m}+\left[\sum_{r=1}^{N}\left(K_{r}-K_{m}\right)V_{r}\cdot \langle A^{\left(r\right)}\rangle \right]{\left[V_{m}+\sum_{r=1}^{N}V_{r}\cdot \langle A^{\left(r\right)}\rangle \right]}^{-1}$$

where *k*_r_ is the thermal conductivity of inclusion phase *r*, and *K*_r_ is the permeability of inclusion phase *r*.

Therefore, the contribution of the present study lies in the integration strategy rather than in the development of new theoretical formulations. Specifically, the DTW-corrected hydration evolution is coupled with the established Mori–Tanaka homogenization theory to form a unified computational framework linking hydration time, phase evolution, and time-dependent effective transport properties.

**Figure 5:**
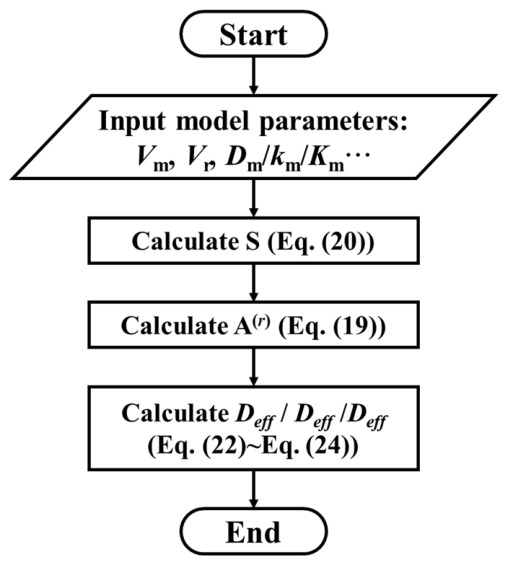
Model solution flowchart.

In summary, the solution procedure of the proposed model is shown in Figure 5.

First, the hydration microstructures at different hydration ages are obtained based on the DTW-CEMHYD3D hydration kinetics model, and the phase volume fractions are statistically determined. Then, the matrix phase and inclusion phases are identified, and the intrinsic transport parameters and shape factors of each phase are specified. Finally, the effective diffusion coefficient, effective thermal conductivity, and effective permeability are calculated based on Mori–Tanaka homogenization.

## 4. Model Validation

It should be noted that the validation cases presented in this section serve different purposes. Cases 1 and 3 compare the proposed framework with independent experimental measurements to evaluate its predictive capability, whereas Cases 2 and 4 mainly compare the proposed model with well-established theoretical models to verify the correctness of the mathematical implementation and the consistency of the homogenization procedure.

### 4.1. Case 1: Validation of the DTW-CEMHYD3D Model

To verify the effectiveness of the DTW-CEMHYD3D hydration kinetics model, the hydration heat prediction results of the classical CEMHYD3D model and the DTW-CEMHYD3D model were compared. The experimental results were taken from Ref. [25].

**Figure 6:**
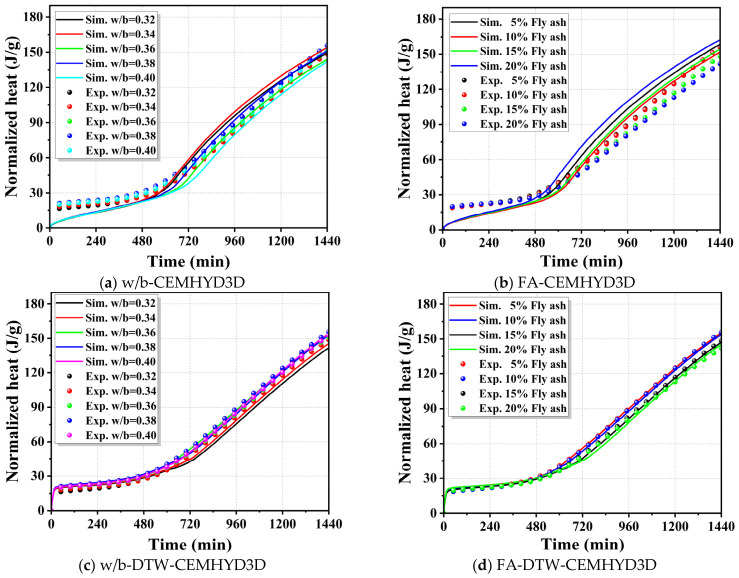
Comparison of CEMHYD3D and DTW-CEMHYD3D simulation results with experimental results.

Figure 6 shows the simulated hydration heat results of the classical CEMHYD3D model and the DTW-CEMHYD3D model for cementitious systems with different water-to-binder ratios and fly ash contents, together with the corresponding experimental results. It can be seen that the classical CEMHYD3D model exhibits obvious deviations from the experimental curves during early-age hydration, whereas the agreement between the simulated and experimental curves is significantly improved after DTW correction. This indicates that the DTW method can effectively correct the correspondence between the simulated hydration time and the real hydration time.

**Figure 7:**
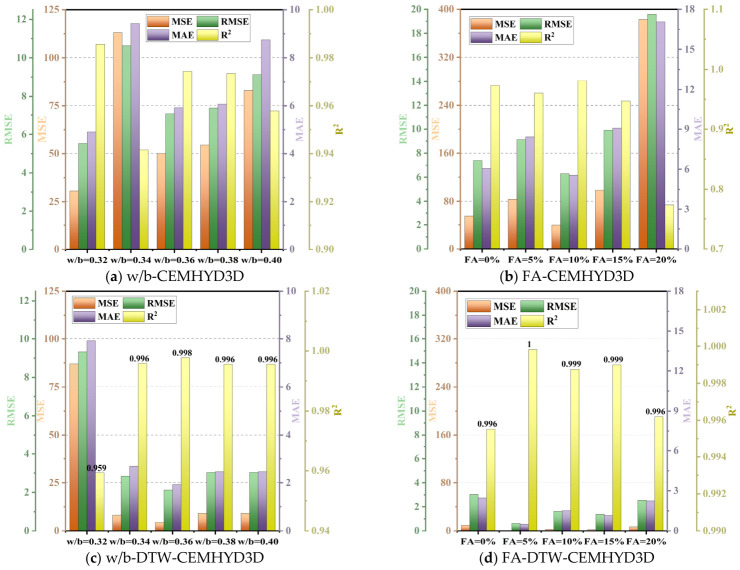
Error analysis of the simulation results from CEMHYD3D and DTW-CEMHYD3D.

Figure 7 further presents the error analysis results of the two models. Compared with the classical model, the DTW-CEMHYD3D model shows an overall reduction in prediction error and a more concentrated error distribution. This indicates that the proposed correction method not only improves the fitting accuracy of the hydration heat curves, but also enhances the adaptability of the model to the early-age hydration process of cementitious systems with different mix proportions.

### 4.2. Case 2: Validation of the Effective Diffusion Coefficient

**Figure 8:**
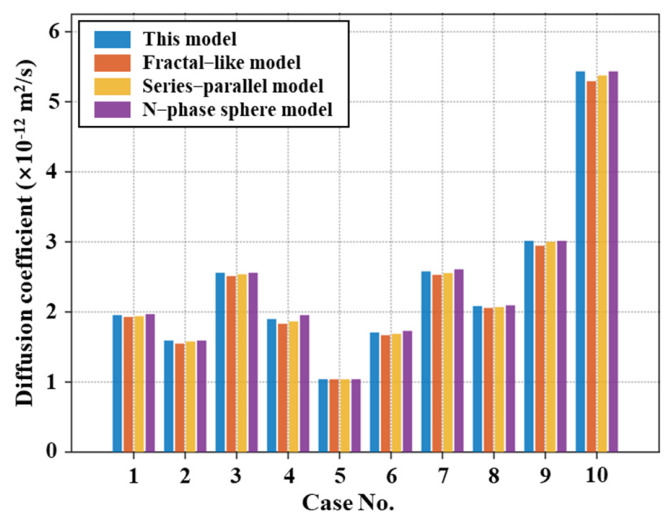
Computational results of four models for 10 types of diffusion coefficients.

To verify the accuracy of the proposed model, three ionic diffusion coefficient models were selected for comparison, namely the series-parallel model [47], the fractal-like model [48], and the N-phase sphere model [49]. The calculated results of different models are listed in Table 1, and the detailed comparison is shown in Figure 8.

As shown in Figure 8, the calculation results of the proposed model are highly consistent with those of the three reference models for the 10 cases, and the overall trends are nearly identical. Among them, the proposed model shows the highest agreement with the N-phase sphere model, with an average relative error of only 0.67%, an R^2^ value of 0.9996, a maximum error of 2.90% in Case 7, and an RMSE of only 0.02 × 10^−12^ m^2^/s. Compared with the series-parallel model, the average relative error is 0.86%, the R^2^ value is 0.9995, and the maximum error is 1.80% in Case 4, indicating excellent consistency. Compared with the fractal-like model, the average relative error is 2.04%, the R^2^ value is 0.9969, and the maximum error is 3.66% in Case 4. Although the relative error is slightly higher, all differences are less than 5%, and these differences mainly result from the different theoretical assumptions and physical mechanisms adopted in the models.

It should be emphasized that the comparison with the N-phase sphere model is not intended as an independent experimental validation. Since both models are developed within the homogenization framework, the excellent agreement primarily demonstrates the correctness of the mathematical implementation and the consistency of the proposed multiphase homogenization procedure. The physical predictive capability of the proposed framework is further assessed through comparisons with independent experimental data in Cases 1 and 3.

### 4.3. Case 3: Validation of the Effective Thermal Conductivity

**Figure 9:**
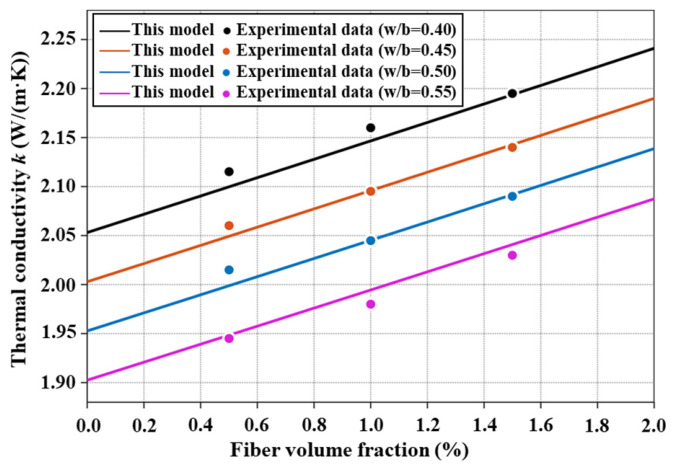
Thermal conductivity test and model validation of fiber-reinforced concrete (experimental data: Ref. [50]).

**Figure 10:**
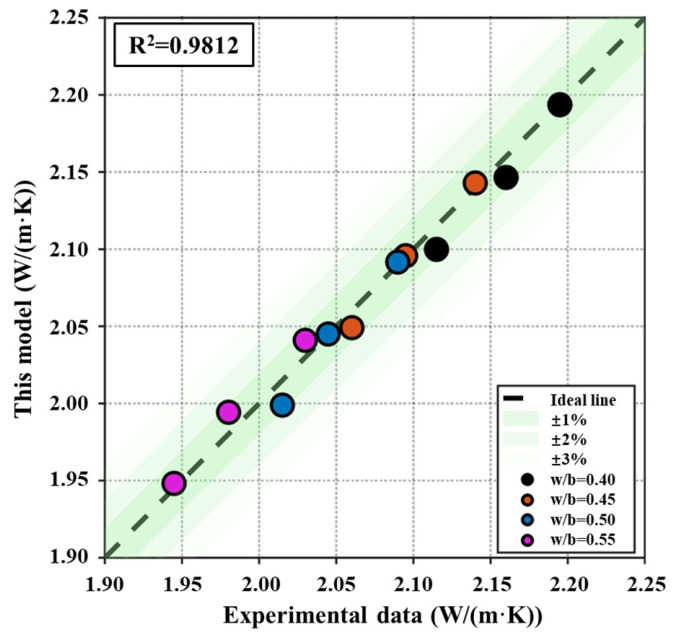
Correlation analysis between measured and modeled thermal conductivity of fiber-reinforced concrete.

To verify the calculation accuracy of the proposed model for the effective thermal conductivity of concrete, the experimental thermal conductivity data of fiber-reinforced concrete reported by Liu et al. [50] were selected for model validation. Although the subsequent analyses in this study mainly focus on plain cement-based materials, the fiber-reinforced concrete case is adopted here to verify the general applicability of the proposed homogenization framework. Since fiber inclusions possess a much higher aspect ratio than hydration products, this validation provides a more rigorous assessment of the model in dealing with inclusion geometry and interfacial effects. Therefore, the agreement with the experimental results supports the applicability of the proposed framework to more conventional cement-based systems. The fiber dimensions were *l* = 30 mm and *d* = 0.05 mm, with *κ* = 60 and *Q* = 1/2. The interfacial effect in fiber-reinforced concrete was considered. The thermal conductivities of the matrix and inclusion phases were taken as *k*_m_ = −1.004 *w/c* + 3.226 W/(m·K), *k*_rITZ_ = 0.65 *k*_m_, and *k*_rf_ = 20 W/(m·K), respectively, and the interfacial transition zone thickness was set as *t*_ITZ_ = 50 nm. The comparison between the model predictions and experimental results is shown in Figure 9 and Figure 10.

The comparison between the model predictions and experimental data shows that the *R*^2^ between the proposed model and the experimental data reaches 0.9812. The average relative error for 12 cases with different fiber contents and water-to-binder ratios is only 0.369%, and the relative errors of all samples are less than 1%. As shown in the scatter plot of predicted and experimental values in Figure 10, the data points are closely distributed near the ideal prediction line and all fall within the ±2% error band, further verifying the reliability of the model.

### 4.4. Case 4: Validation of the Time-Dependent Relative Diffusion Coefficient During Hydration

**Figure 11:**
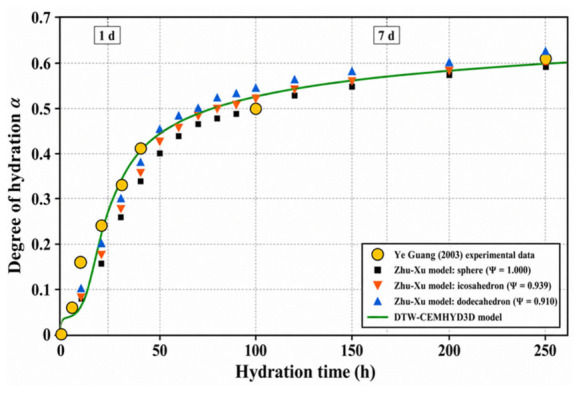
Prediction and validation of hydration degree during continuous hydration process (experimental data: Ref. [51]; model data: Ref. [52]).

Taking the chloride diffusion coefficient as an example, a complete model validation process from hydration evolution to heat and mass transport coefficients was established. The experimental data reported by Ye et al. [51] and the continuum-based HYD-NSP hydration kinetics model developed by Zhu and Xu et al. [52] were selected to validate the accuracy of the proposed model in predicting the degree of hydration and the time-dependent diffusion coefficient. The hydration degree prediction reported by Zhu and Xu et al. [52] was based on the experimental data of Ye et al. [51] for cement paste with a w/b of 0.40 over a hydration period from 0 h to 250 h. Figure 11 first shows the comparison between the hydration degree predicted by the DTW-CEMHYD3D hydration kinetics model and the reference data.

**Figure 12:**
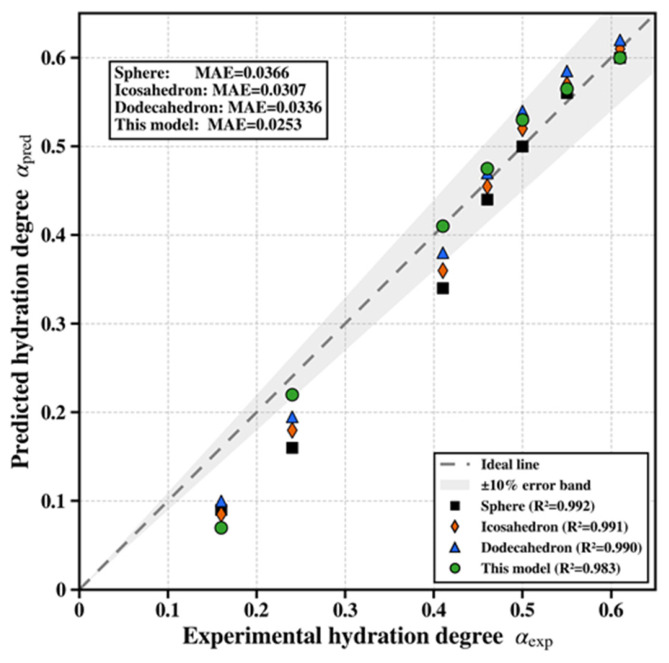
Correlation analysis between measured and modeled degree of hydration.

The results show that the proposed model has an average absolute error of 0.0253 at seven key time points, namely 10 h, 20 h, 40 h, 60 h, 100 h, 150 h, and 250 h. This accuracy is better than that of the Zhu–Xu model [52] under the spherical, icosahedral, and dodecahedral particle morphology assumptions, with the error reduced by 17.6–30.9%. The coefficient of determination of the proposed model reaches 0.9834. In particular, the relative error at 40 h is only 0.4%, while those at 150 h and 250 h are +2.1% and −1.8%, respectively, indicating that the predicted curve agrees well with the experimental data. Meanwhile, Figure 12 shows that the predicted values of the proposed model are evenly distributed on both sides of the ideal 1:1 line, without obvious systematic deviation.

**Figure 13:**
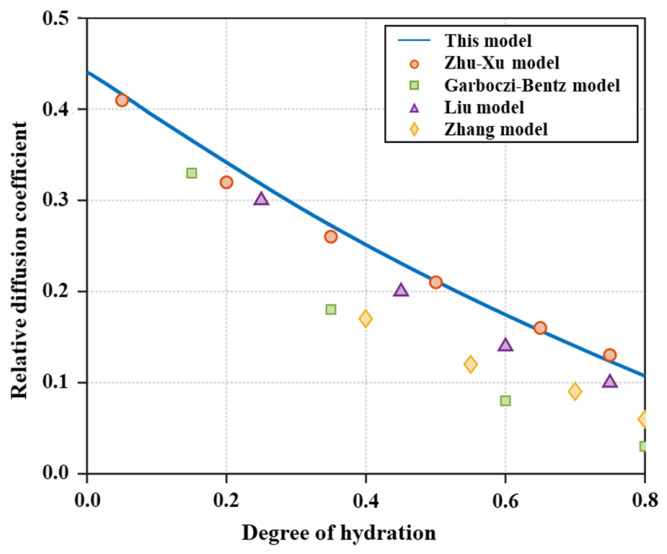
Verification of the relationship between relative chloride ion diffusion coefficient and degree of hydration (data from the Zhu–Xu model [52], Garboczi–Bentz model [53], Liu model [54], Zhang model [26]).

On this basis, the relationship between the relative diffusion coefficient and the degree of hydration obtained from the Zhu–Xu model [52], Garboczi–Bentz model [53], Liu model [54], and Zhang model [26] was further used to validate the diffusion coefficient predicted by the proposed model during hydration. Cement paste with a w/b of 0.50 was selected, and the degree of hydration ranged from 0 to 1. The scalar shape factor used in the homogenization calculation was taken as *Q* = 0.5, following the assumption adopted in the reference comparison. It should be noted that, unlike the models in the literature, DTW-CEMHYD3D is a discrete-based hydration kinetics model. Its hydration process depends on ion dissolution, diffusion, reaction, and nucleation. When the pore connectivity approaches zero, ion diffusion and reaction nucleation become difficult, and the porosity gradually stabilizes. As a result, fewer valid data are available when the degree of hydration exceeds 0.82. Therefore, only data within the degree of hydration range of 0–0.8 were used in the correlation analysis in Figure 13.

**Figure 14:**
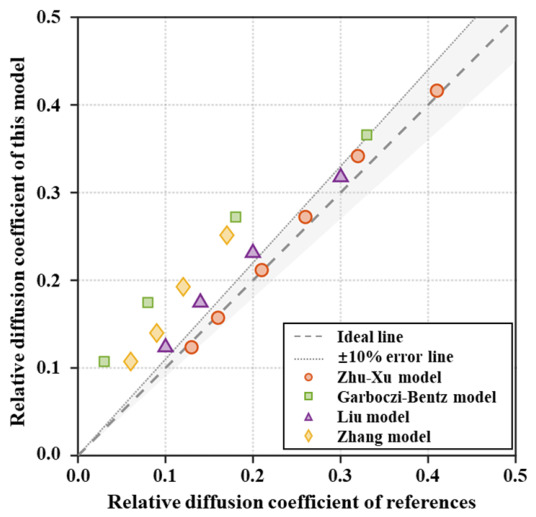
Correlation analysis of relative chloride ion diffusion coefficient and hydration degree.

As shown in Figure 13, the proposed model is consistent with the four reference models in capturing the overall monotonically decreasing trend of the relative diffusion coefficient with increasing degree of hydration. At the early hydration stage, the relative diffusion coefficient ranges from 0.3 to 0.45, and then gradually decreases to 0.03–0.13 as hydration proceeds and reaches a stable stage. This trend is consistent with the physical nature of microstructural densification in cement-based materials during hydration. The correlation analysis results in Figure 14 further quantitatively demonstrate that the proposed model maintains high prediction accuracy compared with the Zhu–Xu model [52], with a coefficient of determination of 0.987. However, certain differences still exist compared with the other models, which mainly arise from different assumptions regarding the microstructure of cement-based materials and the selection of homogenization methods.

## 5. Results and Discussion

### 5.1. Effect of Water-to-Binder Ratio on the Relative Diffusion Coefficient

**Figure 15:**
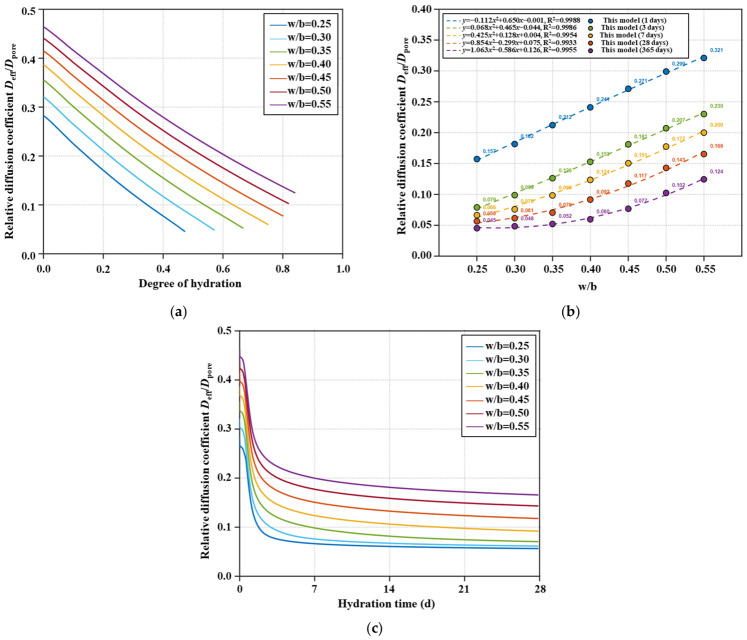
Effect of water–binder ratio and hydration age on the relative diffusion coefficient. (**a**) curve of relative diffusion coefficient vs. degree of hydration; (**b**) curve of relative diffusion coefficient vs. water–binder ratio and hydration time; (**c**) curve of relative diffusion coefficient vs. hydration time.

In this section, the relative diffusion coefficients of cement paste with w/bs ranging from 0.25 to 0.55 were predicted based on the hydration microstructure. The results are shown in Figure 15.

At a hydration age of 1 d, increasing the w/b from 0.25 to 0.55 increases the relative diffusion coefficient from 0.157 to 0.321, corresponding to a growth rate of 104.3%. The fitted relationship between the w/b and the relative diffusion coefficient is D_eff_/D_pore_ = −0.112(w/b)^2^ + 0.650(w/b) − 0.001 (R^2^ = 0.9988). At the critical hydration age of 28 d, the relative diffusion coefficient increases from 0.052 to 0.166. Although the absolute variation is smaller than that at the early age, the growth rate increases sharply to 219.2%, indicating that the w/b has a strong influence on the diffusion coefficient at this age. The fitted equation is D_eff_/D_pore_ = 0.854(w/b)^2^ − 0.299(w/b) + 0.075 (R^2^ = 0.9933), and the quadratic coefficient increases to 0.854. At the long-term hydration age of 365 d, although hydration is nearly complete, the relative diffusion coefficient still increases from 0.045 to 0.124, maintaining a high growth rate of 173.7%. The fitted equation is D_eff_/D_pore_ = 1.063(w/b)^2^ − 0.586(w/b) + 0.127 (R^2^ = 0.9955), and the quadratic coefficient further increases to 1.063. The transition of the quadratic coefficient from negative to positive and its subsequent increase further demonstrate the significant influence of the w/b on the relative diffusion coefficient.

From a microstructural perspective, the water-to-binder ratio affects the transport response mainly by controlling the initial spacing between cement particles and the residual pore connectivity after hydration. A higher water-to-binder ratio introduces more capillary water and leaves a larger fraction of connected pores even after hydration products fill part of the pore space. As a result, the tortuosity of the diffusion pathway is reduced and the relative diffusion coefficient remains higher. This explains why the influence of the water-to-binder ratio becomes more pronounced at later ages, when the difference in residual connected porosity dominates the transport response.

### 5.2. Effect of Fly Ash Content on the Relative Diffusion Coefficient

**Figure 16:**
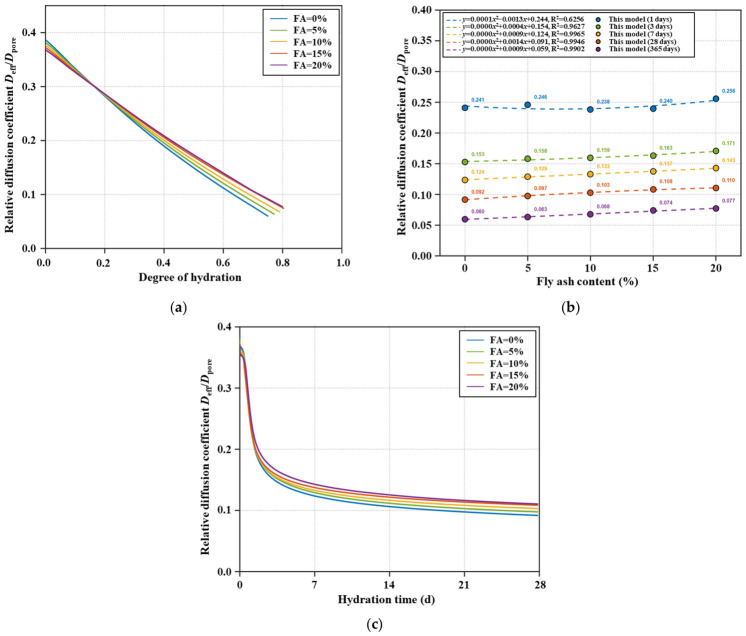
Effect of fly ash content and hydration age on the relative diffusion coefficient. (**a**) Curve of relative diffusion coefficient vs. degree of hydration; (**b**) curve of relative diffusion coefficient vs. fly ash content and age; (**c**) curve of relative diffusion coefficient vs. hydration time.

In this section, the relative diffusion coefficients of blended cementitious systems with fly ash contents ranging from 0% to 20% were predicted based on the hydration microstructure. The results are shown in Figure 16.

At 1 d, increasing the fly ash content from 0% to 20% increases the relative diffusion coefficient from 0.239 to 0.256, corresponding to a growth rate of 7.1%. At 7 d, the relative diffusion coefficient increases from 0.124 to 0.143, and the relative growth rate rises to 15.3%. At 28 d, the relative diffusion coefficient increases from 0.091 to 0.109, with a relative growth rate of 19.8%, which is higher than the 18.5% increase in porosity. This may be attributed to the fact that, at this stage, hydration products formed around fly ash particles create interfacial transition zones with relatively high porosity, which can serve as preferential diffusion paths and lead to a greater increase in the diffusion coefficient than in porosity. At the long-term age of 365 d, the relative diffusion coefficient increases from 0.059 to 0.077.

It can be seen that the sensitivity coefficient of fly ash content to porosity and diffusion coefficient follows a two-stage pattern, shifting from rapid growth to stabilization, with the transition occurring between 7 d and 28 d. This is distinctly different from the nonlinear accelerated differentiation observed for the w/b effect. The linear prediction model established in this section, φ (FA, *t*) = k(t) × FA + φ_0_(t), achieves a prediction accuracy within ±5% of the measured values at 7 d and later ages, with (R^2^ > 0.97), providing a reliable basis for the quantitative optimization of fly ash content.

The age-dependent influence of fly ash originates from the evolution of its pozzolanic reaction. At early ages, fly ash mainly acts as an inert filler and dilution component, resulting in relatively limited changes in the transport properties. As hydration proceeds, the pozzolanic reaction gradually consumes calcium hydroxide and generates additional C-S-H gel, refining the pore structure and reducing pore connectivity. Consequently, the influence of fly ash becomes progressively more significant with increasing hydration age.

It should be noted that the influence of fly ash on chloride transport is not limited to the volumetric change in hydration products and pores. From a cement-chemistry perspective, the pozzolanic reaction of fly ash consumes Ca(OH)_2_ and produces secondary C-S-H, which can refine the pore structure and modify the surface adsorption capacity of the solid phases. In addition, the aluminous components associated with fly ash may affect the formation of AFm phases and hence the chemical binding of chloride ions in the form of Friedel’s salt. Therefore, the age-dependent influence of fly ash on the relative diffusion coefficient should be interpreted as the combined result of microstructural refinement and chemical changes in the binder system. In the present model, these chemical effects are mainly reflected indirectly through the simulated phase volume fractions, whereas explicit chloride-binding reactions are not yet included.

### 5.3. Effect of Pore Saturation Degree on Heat and Mass Transport Coefficients

In actual service environments, liquid and gas phases usually coexist in the pores of cement-based materials. Changes in the pore saturation degree alter the composition of the pore phase and further affect the actual heat and mass transport coefficients of the material. Therefore, based on the multiphase inclusion homogenization model established in Section 3, this section further analyzes the effects of pore saturation degree on the chloride diffusion coefficient and thermal conductivity of cement-based materials.

Let the total pore volume fraction be *V*_p_ and the pore saturation degree be *S*_w_. The volume fractions of liquid-filled pores and gas-filled pores can then be expressed as follows:

(33) $$\begin{array}{l} V_{L}=S_{w}V_{p}\\ V_{G}=\left(1-S_{w}\right)V_{p} \end{array}$$

where *V*_p_ is the total pore volume fraction, which is obtained from the DTW-CEMHYD3D model; *V*_L_ is the volume fraction of liquid-filled pores; and *V*_G_ is the volume fraction of gas-filled pores. As the degree of saturation increases, the proportion of liquid-filled pores gradually increases, whereas that of gas-filled pores gradually decreases.

In this study, cement paste with a w/b of 0.40 and a cement-fly ash composite system with 20% fly ash content (FA20%) are selected as representative cases to analyze the effects of saturation degree on the diffusion coefficient and thermal conductivity, respectively.

#### 5.3.1. Effect of Pore Saturation Degree on the Diffusion Coefficient

**Figure 17:**
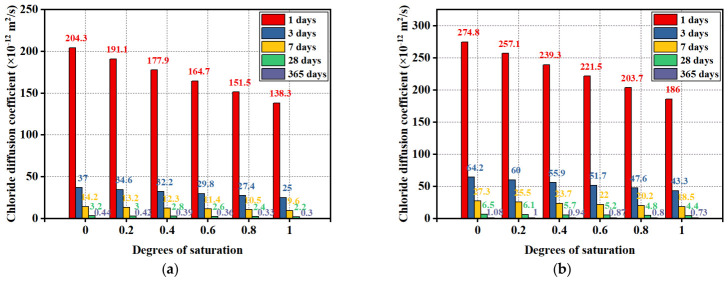
Chloride diffusion coefficients of cement-based materials at different degrees of saturation. (**a**) Chloride diffusion coefficient of paste at a water-to-cementitious ratio of 0.40; (**b**) chloride diffusion coefficient of paste with 20% fly ash replacement.

Figure 17 shows the variation in chloride diffusion coefficients of the cement paste with a w/b of 0.40 and the FA20% composite system under different pore saturation degrees. As shown in the figure, when the combined effects of the solid matrix, liquid-filled pores, and gas-filled pores are considered in the multiphase system, the effective chloride diffusion coefficients of both materials decrease with increasing pore saturation degree.

For the cement paste with a w/b of 0.40 at 365 d, when the pore saturation degree increases from 0 to 1.0, the chloride diffusion coefficient decreases from 0.44 × 10^−12^ m^2^/s to 0.30 × 10^−12^ m^2^/s. For the FA20% composite system at the same age, the chloride diffusion coefficient decreases from 1.08 × 10^−12^ m^2^/s to 0.73 × 10^−12^ m^2^/s. Both materials exhibit clear sensitivity to saturation degree, indicating that changes in the proportions of liquid-filled and gas-filled pores significantly affect the transport capacity of chloride ions in the pore system.

This variation is mainly related to the different transport characteristics assigned to the liquid-filled and gas-filled pore phases in the homogenization model. It should be noted that the intrinsic transport parameters adopted in the present study are effective transport parameters used within the Mori–Tanaka homogenization framework, rather than the actual diffusion coefficients of chloride ions in the corresponding physical media. Therefore, the value assigned to the gas-filled pore phase does not imply physical chloride ion diffusion through air, nor does it represent aerosol or salt-mist transport. Instead, the gas-filled pore phase is introduced as an equivalent constituent to characterize its contribution to the overall transport behavior of the multiphase pore system. Consequently, the predicted dependence of the effective diffusion coefficient on the degree of saturation reflects the combined influence of the relative volume fractions and transport characteristics of different pore phases within the homogenization framework.

Accordingly, the predicted saturation dependence should be interpreted as the response of the equivalent homogenized transport coefficient, rather than the intrinsic chloride diffusion behavior in individual pore phases.

#### 5.3.2. Effect of Pore Saturation Degree on Thermal Conductivity

It should be noted that the thermal conductivity discussed in this section corresponds to hydrated cement paste predicted by the DTW-CEMHYD3D model, whereas the validation case presented in Section 4.3 is based on fiber-reinforced concrete. Since concrete contains aggregates and fibers with much higher thermal conductivity than the cement paste matrix, its overall thermal conductivity is naturally higher. Therefore, the difference in thermal conductivity levels between Section 4.3 and Section 5.3.2 originates from the different material systems rather than any inconsistency in the proposed model.

**Figure 18:**
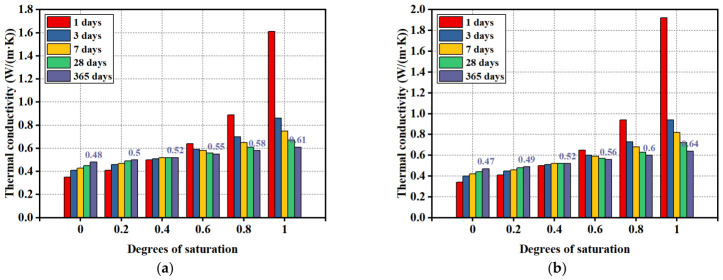
Thermal conductivities of cement-based materials at different degrees of saturation. (**a**) Thermal conductivity of paste at a water-to-cementitious ratio of 0.40; (**b**) thermal conductivity of paste with 20% fly ash replacement.

Figure 18 shows the variation in thermal conductivity of the cement paste with a w/b of 0.40 and the FA20% composite system under different pore saturation degrees. Unlike the chloride diffusion coefficient, the thermal conductivity of cement-based materials slightly increases with increasing pore saturation degree, but the overall variation remains limited.

For the cement paste with a w/b of 0.40 at 365 d, when the pore saturation degree increases from 0 to 1.0, the thermal conductivity increases from 0.48 W/(m·K) to 0.61 W/(m·K). For the FA20% composite system, the thermal conductivity increases from 0.47 W/(m·K) to 0.64 W/(m·K). It can be seen that the thermal conductivities of both materials increase with increasing saturation degree, but the variation is much smaller than that of the chloride diffusion coefficient, indicating that the influence of pore saturation degree on thermal conductivity is relatively weak. This phenomenon is mainly related to the difference in intrinsic thermal conductivity among the solid matrix, liquid phase, and gas phase.

However, as hydration proceeds, the solid skeleton gradually forms and becomes dominant within the material. For the cement paste with a w/b of 0.40 at 365 d, the solid phase volume fraction has reached a relatively high level, and the continuous solid skeleton becomes the main heat conduction path. As a result, the influence of pore phase variation on the overall thermal conductivity is weakened.

Combining the results for the diffusion coefficient and thermal conductivity, it can be concluded that pore saturation degree affects the heat and mass transport properties of cement-based materials through different mechanisms. For chloride diffusion, saturation degree changes the proportions of liquid-filled and gas-filled pores, thereby significantly affecting the diffusion contribution of the pore phase. Therefore, the diffusion coefficient is more sensitive to changes in saturation degree. For heat conduction, although the thermal conductivity of the liquid phase is much higher than that of the gas phase, the solid skeleton becomes dominant at later hydration ages, which significantly weakens the influence of saturation variation on the overall thermal conductivity. Therefore, in predicting the heat and mass transport properties of cement-based materials, the different roles of pore saturation degree in diffusion and heat conduction processes should be distinguished.

The different sensitivities of diffusion and thermal conductivity to the degree of saturation arise from their distinct transport mechanisms. Chloride diffusion mainly occurs through the interconnected liquid-filled pore network; therefore, even a moderate reduction in saturation significantly decreases the continuity of the diffusion pathways. In contrast, heat can be transferred simultaneously through solid phases, liquid-filled pores, and gas-filled pores. As a result, variations in saturation produce a comparatively smaller influence on the effective thermal conductivity.

Although the present framework is established for cement paste, its homogenization strategy is not limited to paste systems. For concrete, fine aggregates, coarse aggregates, and the interfacial transition zone (ITZ) can be explicitly treated as additional inclusion phases within the Mori–Tanaka homogenization framework, and their corresponding transport properties can be incorporated into the multiscale calculation. In this way, the proposed framework has the potential to be extended from cement paste to realistic concrete materials. However, since the present study focuses on hydration-driven microstructural evolution in cement paste, the explicit modeling of aggregates and ITZ is beyond the scope of this work and will be considered in future studies.

From an engineering perspective, this difference indicates that durability-related transport parameters should be selected according to the dominant degradation mechanism: chloride ingress is more sensitive to changes in pore-phase connectivity, whereas heat-transfer analysis is more strongly controlled by the continuity of the solid skeleton at later hydration ages.

## 6. Limitations and Outlook

It should be noted that the homogenization calculation in this study is mainly based on the phase volume fractions obtained from the hydration model, with appropriate simplifications made for detailed structural features such as pore connectivity and interfacial transition zones. In addition, chloride binding by AFm phases and C-S-H, pore-solution chemistry, and the detailed pozzolanic reaction chemistry of fly ash are not explicitly incorporated, and their effects are currently represented only indirectly through changes in phase volume fractions and pore structure. Furthermore, the predicted transport coefficients are inherently influenced by uncertainties in the hydration evolution, phase volume fractions, intrinsic transport properties of individual phases, and the idealized inclusion geometry adopted in the homogenization framework. Although these uncertainty effects are not quantitatively evaluated in the present study, they should be considered when applying the proposed framework to different material systems or service conditions.

Overall, this treatment can meet the requirements for predicting the time-dependent heat and mass transport coefficients in this study, but it can be further extended for complex service environments.

## 7. Conclusions

This study focused on the cross-scale relationship between hydration microstructure evolution and heat and mass transport properties of cement-based materials. A multiscale prediction method combining the DTW-CEMHYD3D hydration kinetics model with Mori–Tanaka homogenization theory was established. On this basis, the effects of water-to-binder ratio, fly ash content, and pore saturation degree on heat and mass transport coefficients were further analyzed. The main conclusions are as follows:

(1) The correspondence between the simulation cycle number in CEMHYD3D and the actual hydration time was corrected using the DTW method, which improved the deviations of the classical CEMHYD3D model in predicting hydration heat during the early dissolution and induction periods.
(2) The proposed model shows good agreement with experimental results and predictions from various classical calculation models. Specifically, in the validation of effective thermal conductivity, the predicted values agree well with the experimental results, with a coefficient of determination of 0.9812 and an average relative error of only 0.369%. In the hydration process validation, the coefficient of determination reaches 0.9834 for the predicted degree of hydration and 0.987 for the predicted relative diffusion coefficient.
(3) The water-to-binder ratio and fly ash content have clear effects on the relative diffusion coefficient. A higher water-to-binder ratio increases residual pore connectivity and thus enhances the relative diffusion coefficient. The influence of fly ash content is age-dependent, mainly because fewer hydration products are formed at early ages, whereas continued hydration and fly ash reaction gradually refine the pore structure at later ages.
(4) Predictive relationships among the relative diffusion coefficient, water-to-binder ratio, hydration age, and fly ash content were established. These relationships enable the relative diffusion coefficient to be determined according to mixture parameters and curing age, providing a practical parameter-input method for chloride transport analysis, durability assessment, and multiphysics coupling simulations of cement-based materials.
(5) Pore saturation degree affects diffusion and heat conduction through different mechanisms. For chloride diffusion, increasing saturation changes the relative contributions of liquid-filled and gas-filled pore phases in the homogenized pore system, leading to a decrease in the predicted effective diffusion coefficient in this study. For heat conduction, the influence of saturation remains relatively limited because the solid skeleton dominates heat transfer at later hydration ages.

## Figures and Tables

**Table 1:** Comparison of calculated chloride diffusion coefficients.

No.	*D*_m_/*D*_r_ ×10^−12^ m^2^/s	*V*_m_/*V_r_*	Fractal-like Model *D* ×10^−12^ m^2^/s	Series-Parallel Model *D* ×10^−12^ m^2^/s	N-Phase Sphere Model *D* ×10^−12^ m^2^/s	This Model *D* ×10^−12^ m^2^/s
1	1/2.1	0.1/0.9	1.92917	1.94033	1.96949	1.95498
2	2.65/1.11	0.4/0.6	1.54823	1.5799	1.59220	1.59220
3	3.41/1.89	0.5/0.5	2.51160	2.53868	2.56017	2.56017
4	0.9/2.33	0.22/0.78	1.83201	1.86536	1.95567	1.89900
5	1.11/0.99	0.43/0.57	1.03957	1.03993	1.04044	1.04044
6	0.98/2	0.23/0.77	1.66878	1.68785	1.73007	1.70736
7	1.8/3.3	0.42/0.58	2.52992	2.55473	2.60930	2.57910
8	1.3/2.3	0.18/0.82	2.05708	2.06953	2.09572	2.08382
9	5.3/2.3	0.31/0.69	2.94621	3.00167	3.01538	3.01538
10	7.7/3.7	0.51/0.49	5.29350	5.37519	5.43384	5.43384

## Data Availability

The original contributions presented in the study are included in the article, further inquiries can be directed to the corresponding author.
